# Nitrate and ammonia as nitrogen sources for deep subsurface microorganisms

**DOI:** 10.3389/fmicb.2015.01079

**Published:** 2015-10-15

**Authors:** Heini Kutvonen, Pauliina Rajala, Leena Carpén, Malin Bomberg

**Affiliations:** ^1^Material Recycling and Geotechnology, VTT Technical Research Centre of FinlandEspoo, Finland; ^2^Materials Performance, VTT Technical Research Centre of FinlandEspoo, Finland

**Keywords:** nitrate reducer, ammonia oxidizer, stable isotope probing, nitrogen cycle, terrestrial deep biosphere, groundwater, crystalline bedrock, nitrogen assimilation

## Abstract

We investigated the N-utilizing bacterial community in anoxic brackish groundwater of the low and intermediate level nuclear waste repository cave in Olkiluoto, Finland, at 100 m depth using ^15^N-based stable isotope probing (SIP) and enrichment with ^14∕15^N-ammonium or ^14∕15^N-nitrate complemented with methane. Twenty-eight days of incubation at 12°C increased the concentration of bacterial 16S rRNA and nitrate reductase (*nar*G) gene copies in the substrate amended microcosms simultaneously with a radical drop in the overall bacterial diversity and OTU richness. *Hydrogenophaga/Malikia* were enriched in all substrate amended microcosms and *Methylobacter* in the ammonium and ammonium+methane supplemented microcosms. *Sulfuricurvum* was especially abundant in the nitrate+methane treatment and the unamended incubation control. Membrane-bound nitrate reductase genes (*nar*G) from *Polarimonas* sp. were detected in the original groundwater, while *Burkholderia, Methylibium*, and *Pseudomonas nar*G genes were enriched due to substrate supplements. Identified *amo*A genes belonged to *Nitrosomonas* sp. ^15^N-SIP revealed that Burkholderiales and Rhizobiales clades belonging to the minority groups in the original groundwater used ^15^N from ammonium and nitrate as N source indicating an important ecological function of these bacteria, despite their low number, in the groundwater N cycle in Olkiluoto bedrock system.

## Introduction

Nitrogen (N) is one of the basic elements of all life forms and is essential for the production of amino and nucleic acids (Bothe et al., 2007). N depletion may be a growth-limiting factor in many environments, such as deep bedrock groundwater. However, use of explosives in e.g., construction into bedrock environments and mining may increase the concentration of N compounds in these environments, due to under detonated explosives (Beller et al., 2004). Use of pesticides, such as atrazine, has also been shown to be a source of N pollution in karst aquifers that are in connection to agricultural sites (Iker et al., 2010). Despite the generally low metabolic activity of deep groundwater microorganisms (e.g., D'Hondt et al., 2002; Rajala et al., 2015a) these N compounds may affect the activity and growth of deep bedrock microbial communities.

Nitrate is generally present only at low concentrations in pristine anoxic crystalline bedrock groundwater because it has been used as an alternative electron acceptor to oxygen and reduced to N_2_ in anaerobic redox processes (Bothe et al., 2007; Posiva, 2009). Dissimilatory nitrate reduction is a process where the oxidative force of nitrate is used as energy source in the oxidation of organic substrates by denitrifying bacteria. Denitrifying bacteria are mostly heterotrophic and often facultatively anaerobic with the ability to switch between oxygen and nitrate respiration depending on the environmental conditions (Luque-Almagro et al., 2011). Nitrate-reducing bacteria (NRB) have been shown by MPN enrichment studies to colonize groundwater in Olkiluoto, Finland at least to a depth of 300 m (Pedersen, 2008). The same study showed that the distribution and abundance of NRB correlated with that of the cultivable heterotrophic aerobic bacteria, indicating that nitrate as an optional terminal electron acceptor if oxygen is not available. However, depending on depth of the groundwater and location of the study site, denitrification and nitrate respiration in deep crystalline bedrock groundwater have been predicted to be low based on studies focusing on detection of essential genes for denitrification in deep crystalline bedrock environments (Nyyssönen et al., 2014; Purkamo et al., 2015). Nevertheless, NRB otherwise below the detection limit have been shown to significantly increase the transcription of *nar*G genes in response to increased concentration of methane together with sulfate under N_2_ atmosphere in deep groundwater (Rajala et al., 2015a). In addition, it has been shown that certain ε-proteobacterial lineages couple reduction of nitrate to simultaneous oxidation of sulfide (reviewed in Campbell et al., 2006).

Ammonia/ammonium is an essential nutrient and energy source in both oceanic and terrestrial hydrothermal environments, where genes involved in ammonia oxidation have been found (Wang et al., 2009). However, little is known about the microbial community metabolizing ammonia or ammonium in pristine deep and cold crystalline bedrock groundwater. In the nitrification process ammonia is oxidized via nitrite to nitrate by the ammonia monooxygenase (AMO) enzyme. In pristine groundwater the concentration of ammonia is low. However, ammonia may be produced during fermentation of biomass, such as dead biofilm and ancient organic material trapped in the rock. In addition, under-detonated explosives may serve as an ultimate source for the production of ammonia by the DNRA process. Nevertheless, ammonia-oxidizing bacteria (AOB) have been found to be scarce in Fennoscandian Shield groundwater (Purkamo et al., 2015).

In addition to functioning as energy sources both nitrate and ammonia are N sources for microorganisms. Nyyssönen et al. (2014) showed by analysing metagenomic data from the Outokumpu deep borehole, that ammonia was the main source of N in the deep groundwater.

The N cycle in deep crystalline bedrock groundwater environments have not yet been extensively studied. In karst-bedrock environment the aerobic and anaerobic oxidation of ammonia has been shown to be slow (Ray et al., 2010) and the microbial communities changed as a result of N contamination by atrazine (Iker et al., 2010). In deep geological repositories for spent nuclear fuel, introduced N compounds may have great implications for the long-term safety of the stored nuclear waste. In Finland, low and intermediate level radioactive waste is stored in a repository at 100 m depth in the bedrock of Olkiluoto Island since 1992. This waste consists of metallic and organic materials from maintenance and decommissioning activities in the nuclear power plant. N compounds, such as ammonium and nitrate may increase microbial activity in this environment, which may cause microbially induced corrosion of the steel and cause release of radionuclides (Rajala et al., 2015b). Here we aimed to gain more insight to both the energy-producing and assimilatory aspects of the N cycle, which could affect the stability of the low and intermediate level radioactive waste repository. We focused especially on nitrate and ammonium in the deep groundwater, since these compounds may be introduced in to the groundwater system at the repository site through construction of the repository. In microcosm experiments we followed the consumption on these N compounds as well as the change in the general bacterial as well as NRB and AOB communities influenced by these compounds. In addition, we aimed to identify the nitrate and ammonium assimilating bacterial community by Stable Isotope Probing (SIP) with ^15^N labeled substrates.

## Materials and methods

### Chemistry

The chemical composition of groundwater in the beginning of the experiment was analyzed at the ALS Finland. Dissolved metals were analyzed by ICP-OES and N compounds spectrophotometrically according to the CSN ISO 11732 and CSN ISO 13395. pH, redox-potential, oxygen content and temperature of the water were measured inside an anaerobic glove bag with a HACH Sension 156 m (Hach Lange Gmbh, Germany) and electrical conductivity with a CDM92 conductivity meter (Radiometer-analytical, France) on site.

### Experimental setup

Groundwater samples were collected in May 2013 from drill hole VLJ-KR9 in the low and intermediate level nuclear waste repository tunnel situated in the crystalline bedrock at the site of the Olkiluoto nuclear power plant, Finland. The drill hole VLJ-KR9 was drilled in 1995 in to the wall of the VLJ-tunnel at approximately 95 m depth from the ground surface. The total length of the drill hole is 20.04 m and it bears slightly downwards reaching a depth of 95.249 m from the ground surface. The first 66 mm of the drill hole is cased and the opening is sealed with a welded flange plug equipped with a pressure sensor and tap for sampling. The drill hole is regularly purged. Before the sampling a sterile, gas tight polyacetate tube was connected to the sampling tap of the borehole and groundwater was run for 30 min before the start of the sample collection in order to remove excess air from the tube.

Altogether 30 groundwater samples of 2.4 L each were collected in sterile borosilicate glass bottles previously rendered anaerobic under constant N_2_ flow. The groundwater was introduced to the bottles through the sterile polyacetate tube directly from the borehole located in the wall of the tunnel. In order to reduce exposure to oxygen in the microcosm bottles a volume of 50% overflow was allowed before each bottle was sealed with a tight-fitting butyl rubber septum and open-top screw-cap. The water samples were transported in a cooling-box with ice to the laboratory within 1d of sampling. The sample bottles were divided into 10 treatment groups with three replicates per treatment. The treatments were; (1) baseline sample, biomass was captured on 0.2 μm pore-size cellulose acetate membranes (Corning, MA, USA) by filtering 2.4 L groundwater on-site immediately after sampling and immediately frozen on dry ice, (2) Na^15^NO_3_ addition, (3) ^15^NH_4_Cl addition, (4) Na^15^NO_3_+CH_4_addition, (5)^15^NH_4_Cl+CH_4_addition, (6) Na^14^NO_3_addition, (7)^14^NH_4_Cl addition, (8) Na^14^NO_3_+CH_4_addition, (9)^14^NH_4_Cl+CH_4_ addition, and (10) no substrate additions (incubation control). N substrates and methane were added at the beginning of the experiment and again after 14 days of incubation. The N substrates (sodium nitrate and ammonium chloride, atom 98% ^15^N, Sigma-Aldrich) were sterilized using a 0.2 μm pore size syringe filter and rendered anaerobic by sterile N_2_ gas flow for 30 min. Five milliliters N substrates (62.5 mM stock solution) were added to a final concentration of 0.13 mM. The N substrates and 5 mL sterile methane (treatments 4, 5, 8, and 9), i.e., approximately 2 mL L^−1^ sample water, were added aseptically to the 2.4 L microcosms through the butyl rubber stoppers using a sterile syringe and needle. The microcosms were incubated at a temperature of +12°C, which was as close to that of the drill hole at 95 m depth from ground surface as possible for 28 d.

The biomass of each sample was collected by vacuum suction on 0.2 μm-pore-size cellulose acetate membranes (Corning, MA, USA) at the end of the incubation period. The filter membranes were cut out of the filter funnels using sterile scalpels, inserted into sterile 50 mL screw cap tubes (Corning, MA, USA) and frozen at −80°C until use.

The consumption or production of nitrate and ammonium was monitored by subsamples retrieved at the beginning of the experiment before and after addition of substrates, after 14 days incubation before and after addition of substrates and at the end of the incubation period. At each sampling time, 5 mL subsamples were retrieved using a sterile syringe and needle pushed through the butyl rubber septum of each bottle. The concentration of nitrate and ammonium of each subsample was measured spectrophotometrically using the Hach–Lange DR 2800 spectrophotometer (Hach–Lange, UK) and the LCK304 kit for ammonium and LCK339 kit for nitrate according to the manufacturer's instructions. The consumption and production of nitrate and ammonium was determined by comparing the concentration of N compounds after 14 and 28d of incubation to the concentrations immediately after substrate addition. The balance of N substrates in the different incubations was normalized to relative abundances of the measured N compounds in each different incubation type for better comparison between different treatments.

### DNA extraction

Filters with the collected biomass were carefully thawed on ice. Microbial DNA was extracted using the Metagenomic isolation kit for water (Epicentre, USA). The DNA was extracted according to the manufacturer's protocol and eluted in 50 μL elution buffer.

### Quantification of the number of bacteria and nitrate reducers by qPCR

The bacterial population in the original groundwater and in the water samples after incubation with or without substrates was determined by 16S rRNA gene targeted qPCR using universal bacterial 16S rRNA gene-targeting primers fD1 (Weisburg et al., 1991) and P2 (Muyzer et al., 1993). In addition, the concentration of *nar*G genes of the nitrate reducers was tested with primers narG1960m2f/narG2050M2F (López-Gutiérrez et al., 2004).

The qPCR reactions were performed in 10 μL reaction volumes using the KAPA 2 × Syrb® FAST qPCR-kit on a LightCycler480 qPCR machine (Roche Applied Science, Germany). Each reaction contained 2.5 μM of relevant forward and reverse primer and 1 μL DNA extract. Each reaction was run in triplicate and no-template control reactions were used to determine background fluorescence in the reactions.

The qPCR conditions consisted of an initial denaturation at 95°C for 10 min followed by 45 amplification cycles of 15 s at 95°C, 30 s at 55°C, and 30 s at 72°C with a quantification measurement at the end of each elongation. A final extension step of 3 min at 72°C was performed prior to a melting curve analysis. This consisted of a denaturation step for 10 s at 95°C followed by an annealing step at 65°C for 1 min prior to a gradual temperature rise to 95°C at a rate of 0.11°C s^−1^ during which the fluorescence was continuously measured. The number of bacterial 16S rRNA genes and *nar*G genes was determined by comparing the amplification result (Cp) to that of a 10-fold dilution series (10^1^–10^7^ copies μL^−1^) of *Escherichia coli* (ATCC 31608) 16S rRNA genes and *Pseudomonas aeruginosa* (ATCC 15692) *nar*G, respectively, in plasmid. A qPCR assay for the *amo*A genes was not conducted due to low specificity of the used primers to the *amo*A gene (see Section Ammonia Oxidizers).

### PCR

PCR reactions for denaturing gradient gel electrophoresis (DGGE) analysis of the bacterial 16S rRNA genes, *nar*G and *amo*A gene profiles in the original groundwater and after the incubation period were performed in 50 μL reactions. The reactions contained 1 u KAPA Hifi—polymerase in 1 × KAPA HF-buffer, 0.6 mM dNTP, 0.4 μM of each relevant primer and 1 μL DNA extract. The PCR program consisted of a 5 min initial denaturation step at 98°C followed by 40 cycles of denaturation at 98°C for 20 s, annealing at 55°C (16S rRNA genes) or 57°C (*amo*A and *nar*G) for 15 s and elongation at 72°C for 30 s, and a final extension at 72°C for 5 min. For the bacterial 16S rRNA genes, primers U968fGC /U1401r (Nübel et al., 1996) were used; the *nar*G and *amo*A DGGE PCR products were obtained with primers *nar*G2179F/*nar*G2488R-GC (Pastorelli et al., 2013) and *amo*A1F/*amo*A2RGC (Nicolaisen and Ramsing, 2002), respectively.

### DGGE

Profiles of bacterial communities having 16S rRNA gene, *nar*G and *amo*A genes were resolved vs. different treatments using DGGE. The DGGE was performed on a Bio-Rad DCode TM Universal Mutation Detection System (Bio-Rad, USA). For the 16S rRNA gene fragments, a denaturing gradient of 35–65% was used and the electrophoresis was run for 20 h at 85 V at 60°C as described in Nübel et al. (1996). The denaturants used was urea (42 g/100 mL in 100% denaturing solution) and formamide (40% formamide in 100% denaturing solution). The denaturing gradient used for the *amo*A-gene fragments was 30–70% and the electrophoresis was run for run 6 h with 200 V at 60°C according to Nicolaisen and Ramsing (2002). The *nar*G-gene fragments were subjected to DGGE using a denaturing gradient of 40–70% for 17 h with 70 V at 60°C according to Pastorelli et al. (2013). All gels were made of 8% polyacrylamide.

DGGE bands were detected using SYRB-green I nucleic acid stain (Fisher Scientific, UK) according to the manufacturers protocol and imaged under UV light using a GEL DOC XR+2000 transilluminator (Bio Rad, USA). Prominent DGGE fragments were extracted from the gels using sterile plastic Pasteur pipettes. DNA from the gel plugs was extracted into 20 μL of molecular grade water overnight at 4°C and the DGGE band was reamplified with the relevant primer pair without the GC-clamp, as described above using 2 μL extracted DNA as template. The reamplified DGGE bands were shipped to Macrogen Inc, South Korea on ice packs, for sequencing.

Phylogenetic analyses were performed on the sequences of the DGGE bands with Geneious Pro (Biomatters Ltd, New Zealand). All sequences were imported into Geneious Pro, and aligned to reference sequences and most closely matching sequences in the NCBI nucleotides database (http://blast.ncbi.nlm.nih.gov/) determined with the blastn tool in Geneious Pro. The alignments were performed with the Mafft tool in Geneious Pro with default settings and the alignments were edited manually. Phylogenetic analyses were performed on the alignments of the 16S rRNA gene sequences using PhyML (Guidon and Gascuel, 2003) with the Jukes-Cantor69 substitution model (Jukes and Cantor, 1969). The *nar*G and *amo*A gene sequences were translated in to amino acid sequences using translation table 11 prior to alignment with Mafft and a phylogenetic analysis with PhyML using the WAG substitution model (Whelan and Goldman, 2001). Bootstrap support for nodes were calculated based on 1000 random repeats for all phylogenetic analyses.

### Isopycnic centrifugation and gradient fractionation

In order to separate the ^15^N-containing DNA fraction of the microbial community that had consumed the N substrates from the rest of the community DNA density gradient centrifugation was performed using 5 ml Quick-seal polyallomer tubes (BeckmanCoulter, Brea, CA, USA) in a Beckman ultracentrifuge with a VTI 65.2 vertical tube rotor. Each tube contained a fixed volume of 35 μL extracted DNA suspended in 4.9 mL of CsCl in gradient buffer (5 mL 1 M Tris-HCl, 0.375 g KCl, 0.15 mL EDTA) with a density of 1.725. The DNA was centrifuged for 62 h at 44,100 g at 22°C.

After centrifugation the gradients were apportioned, starting at tube bottoms, into 22 equal fractions of approximately 200 μL. The centrifugation tube was first pierced with hypodermic needles at the top and bottom and the fractions were pushed out through the bottom needle by pushing sterile water mixed with dye at a rate of 200 μL min^−1^ in to the top of the centrifuge tube using a syringe pump (New Era Pump Systems, Inc., Farmingdale, NY, USA). Gradient formation in the centrifugation was determined by including a control tube without DNA in each run and fractioning the gradient as for the DNA samples. The nd value of each of the control fractions was determined using a refractometer (DR301-95, A. Krüss Optitronic, Germany). In addition, the nd value of the first and last fraction of the DNA sample tubes was also measured in order to ensure that a gradient has been formed during the centrifugation.

Due to the potential for low separation efficiency when there is low concentration of N in the DNA and the high density of GC-rich DNA, fractions with densities 1.725–1.733 were recentrifuged as before, but with the addition of 8 μL (10 mg mL^−1^) bis-Benzimide in each sample to improve buoyancy of GC-rich DNA as described in Buckley et al. (2007). This was performed according to gradient formation in controls during the first centrifugation. After centrifugation, gradients were apportioned as before directly onto MultiScreenf® Filter Plates (Merck Millipore Ltd., Cork, Ireland) and purified from CsCl salt using a 96-well Vacuum Manifold (Merck Millipore Ltd., Cork, Ireland) with 5 volumes (200 μL) sterile water.

Fractions containing heavy and light DNA were determined by qPCR as described above for the bacterial 16S rRNA gene. DNA from fractions having density of 1.72 and 1.735 were determined to contain DNA fractions of interest and subjected to high throughput (HTP) amplicon sequencing.

### High throughput amplicon sequencing and sequence analysis

Libraries for 454 HTP amplicon sequencing were prepared by PCR of DNA from the original groundwater used in the experiment, the supplemented incubations and incubation control, and also the heavy-fraction DNA of the Na^15^NO_3_,^15^NH_4_Cl, Na^15^NO_3_+CH_4_, and^15^NH_4_Cl+CH_4_ treatments. For HTP sequencing, amplicon libraries from the three replicates of each treatment were combined due to the low amount of DNA in the heavy fraction DNA extracts.

Bacterial 16S rRNA fragments covering the V1–V3 variable regions were amplified with primers 8F (Edwards et al., 1998) and P2 (Muyzer et al., 1993) equipped with adapter and MID sequences (tags) at their 5' end in a single round PCR as described in Bomberg et al. (2015). PCRs were performed with KAPA HiFi polymerase (Kapa Biosystems, Inc., Boston, MA, USA) in 1 × HF buffer. Each 50 μL reaction contained 0.5 mM dNTP and 1 μM primer mix. PCR conditions consisted of an initial denaturation step of 30 s at 98°C, followed by 35 cycles of 10 s at 98°C, 15 s at 55°C, and 15 s at 72°C, and a final extension step at 72°C for 5 min. Sequencing of the PCR products was performed at BeckmanCoulters Genomics using the FLX 454 Titanium (454 Life Sciences, Branford, CT, USA).

Sequence reads were analyzed using Mothur (v 1.33.1, Schloss et al., 2009) where the flow-grams were denoised using the default parameters of the sff.multiple workflow in Mothur, trimmed to remove adapter, barcode, and primer sequences, and to exclude sequences that did not meet the quality criteria (i.e., no barcode and primer mismatches, no ambiguous nucleotides, maximum eight-nucleotide-long homopolymer stretches and defined minimum length of 200 bp). The bacterial 16S rRNA sequences were aligned to the Silva v_119 reference alignment (Pruesse et al., 2007) and the alignments were screened to include sequences with defined start positions and minimum end position. The aligned sequences were preclustered prior to chimera detection with the Chimera.slayer command in Mothur and possible chimeric sequences were removed. A distance matrix was calculated for the chimera-filtered sequences using a cut-off of 0.05 without penalty for end gaps. The sequences were clustered into Operational Taxonomic Units (OTUs) according to the distance matrix using the nearest-neighbor method. The representative sequences of the OTUs sharing 97% sequence similarity within each OTU were classified using the Silva v_119 reference database (Pruesse et al., 2007). Alpha diversity analyses were performed on data normalized to 448 sequence reads per sample and rarefaction analyses were performed on the total number of sequence reads per sample.

### Accession numbers

The sequences of the DGGE bands will be deposited in the European Nucleotide Archives (ENA; https://www.ebi.ac.uk/ena) under accession numbers LN866782-LN866813 for 16S rRNA genes, LN866814-LN866821 for *amo*A genes and LN866822-LN866841 for *nar*G genes. The HTP sequence reads were submitted to ENA under accession numbers ERS739681-ERS739690.

## Results

### Groundwater chemistry

At the time of sampling the groundwater contained 0.178 mg L^−1^ ammonium-N, 0.229 mg L^−1^ ammonium, <0.040 mg L^−1^ nitrate, and <0.150 mg L^−1^ nitrite at pH 8.06. The pH, temperature (T), reduction potential (Eh) and electric conductivity (Ec) of the groundwater was 8.09, 10.3°C, 58 mV vs. Standard Hydrogen Electrode (SHE) and 2.26 mS cm^−1^, respectively. Other physicochemical parameters are presented in Table 1.

**Table 1:** **The physicochemical composition of the groundwater at the time of sampling**.

**Measurement**	**Value**		
T	10.3°C		
O_2_	0.09 mg L^−1^		
Alkalinity	5.28 mmol L^−1^		
Eh	58 mV vs. SHE		
Ec	2.26 mS cm^−1^		
Sulfide	0.061 mg L^−1^		
Phosphate	0.223 mg L^−1^		
CO_2free_	3.68 mg L^−1^		
CO_2total_	236 mg L^−1^		
F_total_	0.378 mg L^−1^		
Br_total_	1.93 mg L^−1^		
Cl_total_	513 mg L^−1^		
Ca _total_	76.3 mg L^−1^	Ca _soluble_	74 mg L^−1^
Mg _total_	20.3 mg L^−1^	Mg _soluble_	21.6 mg L^−1^
Na _total_	421 mg L^−1^	Na _soluble_	420 mg L^−1^
Fe _total_	0.109 mg L^−1^	Fe _soluble_	0.0624 mg L^−1^
Mn _total_	0.161 mg L^−1^	Mn _soluble_	0.153 mg L^−1^
S _total_	40.4 mg L^−1^	S _soluble_	48.7 mg L^−1^

*For the metals Ca, Mg, Na, Fe, Mn, and S both the total (_total_) and the soluble (_soluble_) forms were measured*.

### Consumption of ammonium and nitrate

**Figure 1:**
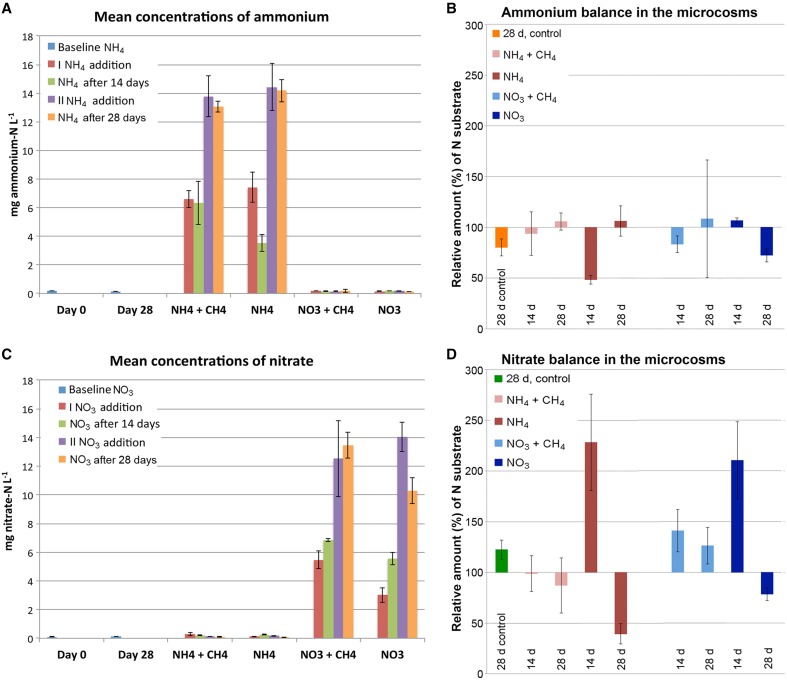
**The average concentration (A,C) and relative balance (B,D) of ammonium and nitrate in the microcosms**. In **(A,C)** the average concentration of ammonia or nitrate (mg L^−1^) is measured from the original groundwater and the 28d controls (blue), directly after the first addition of N-substrate (red), 14d after the first addition of N-substrate (green), directly after the second addition of N-substrate (purple), and at the end of the experiment (orange). In **(B,D)** the relative change in the concentration of ammonium and nitrate is illustrated for incubations with NH_4_+CH_4_ (pink), NH_4_ (red), NO_3_+CH_4_ (light blue) and NO_3_ (dark blue) after 14 and 28d of incubation. The change in ammonium concentration over 28 days in **(B)** is shown in orange and the change in nitrate concentration in **(D)** in green. The relative amount of N substrates in **(B,D)** above 100% are considered produced and below 100% consumed compared to the amount of N substrates measured directly after addition of substrates.

The original concentrations of ammonium and nitrate were low (Figures 1A,C). During 4 weeks of incubation at 12°C the amount of ammonium decreased almost 20% from the original concentration at the time of sampling (Figure 1B). The concentration of nitrate, on the other hand increased 22.5% over the same time period (Figure 1D). After addition of the N substrates, the concentration of ammonia decreased 34.5% in the microcosms that had received ammonium and methane (NH_4_+CH_4_) and 51.5% those only receiving ammonium (NH_4_). However, the ammonium concentration ceased to decrease after 2 weeks of incubation (Figure 1). In the nitrate-supplemented microcosms, the relative concentration of nitrate increased in all treatments after the first addition of nitrate (Figure 1D). However, after 4 weeks of incubation 21.2% of the added nitrate had been consumed in microcosms supplemented with nitrate (NO_3_) only, while the nitrate concentration in the nitrate+methane (NO_3_+CH_4_) remained high.

Over the time of incubation the concentration of nitrate increased with approximately 25% in the unamended control microcosms, while the concentration of ammonium decreased by the same magnitude (Figure 1). The concentration of ammonium in the nitrate-supplemented microcosms increased slightly toward the end of the incubation period in the NO_3_-supplemented microcosms, and during the first 14 days in the NO_3_+CH_4_-supplemented microcosms, but was not extensively produced from the added ammonium (Figure 1B). In the NH_4_+CH_4_-supplemented microcosms, on the other hand, the NO_3_ concentration increased noticeably with approximately 230% during the first 14 days of incubation where after the NO_3_ was consumed to about 40% of the original NO_3_ concentration by the end of the incubation period, indicating that NO_3_ was initially produced from the added NH_4_ (Figure 1D).

### Quantitative analysis of microbial community

**Figure 2:**
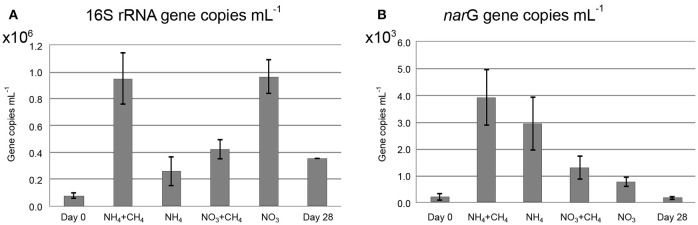
**The concentrations of (A) bacterial 16S rRNA gene copies, (B) ***nar***G gene copies mL^−1^ in the original groundwater (Day 0), the incubations with substrates (NH_4_+CH_4_, NH_4_, NO_3_+CH_3_, NO_3_) and without substrates (Day 28)**.

There were 7.8 × 10^4^ copies mL^−1^ of the bacterial 16S rRNA gene in the original groundwater. The addition of NH_4_+CH_4_ and NO_3_ had the greatest effect on the bacterial community and increased the concentration of bacterial 16S rRNA gene copies 12-fold over the 28d incubation period to 9.5 × 10^5^ mL^−1^ and 9.7 × 10^5^ mL^−1^, respectively (Figure 2A). In the other treatments the number of bacteria also increased, including the control (unamended) microcosms, by three to five fold.

There were 2.2 × 10^2^ *nar*G gene copies mL^−1^ corresponding to the population of NRB in the original groundwater (Figure 2B). The NH_4_+CH_4_ treatment had the greatest effect on the NRB increasing the concentration of *nar*G genes to 3.9 × 10^3^ copies mL^−1^ over the course of incubation. In nitrate-treated microcosms the increase in *nar*G genes was 2.7 × 10^3^ copies mL^−1^. Without substrate additions, the concentration of *nar*G genes did not increase during the incubation time.

### Bacterial community composition

**Figure 3:**
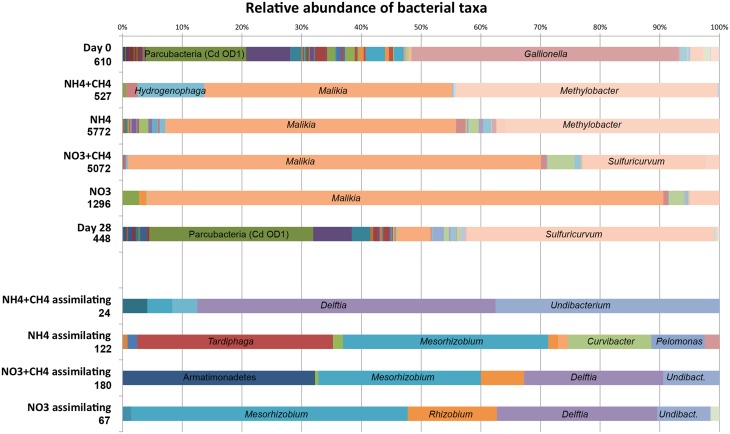
**The relative abundance of bacterial genera detected by high throughput amplicon sequencing from the original groundwater (Day 0), N- and C-supplemented microcosms and the non-supplemented control (Day 28)**. The lower part of the figure shows the relative abundance of nitrogen assimilating bacteria detected from the ^15^N fractions. The number in by each category indicates the number of sequence reads. The names of the most prominent genera are indicated in the bar graph. More detailed information of the relative abundance of different bacterial taxa detected in the study can be found in Table S1.

The number of sequence reads obtained from 454 pyro sequencing ranged from 448 to 5772 sequence reads per sample from the original groundwater and the different microcosms (Table 2). The bacterial community in the original groundwater was diverse (H' = 3.07) according to the 454 amplicon sequence profiles, which showed 125 OTUs belonging to 61 different bacterial genera (Figure 3, Table 2, Table S1). The richness estimates (chao and ace) indicated that only between 21 and 41% of the OTU richness in the original groundwater was captured. Over 28d the microbial OTU diversity increased (H' = 3.51) to altogether 146 OTUs, but the number of identified genera decreased to 43 and according to the richness estimates only 19.2–35.5% of the total OTU richness was obtained. In the substrate amended microcosms the diversity was significantly lower, H' = 0.04–0.70, than that of the original groundwater and the 28d incubation controls. The number of observed OTUs ranged from 2 to 19 and according to the richness estimates between 60.8 and 100% of the total diversity of the bacterial communities was captured (Table 2). The low number of OTUs obtained as well as the low diversity and richness indices indicates that the addition of N substrates enriched a specific population of bacteria involved in N cycling during the incubation.

**Table 2:** **Alpha diversity estimates of the original groundwater (Day 0) and after 28 days of incubation in the control microcosms (Day 28) and the microcosms supplemented with NH_4_+CH_4_, NH_4_, NO_3_+CH_4_, NO_3_**.

**Sample**	**Number of sequences**	**Number of observed OTUs**	Estimation of OTU richness^*^		**% richness captured**		Diversity index^*^	
			**Chao**	**ace**	**chao**	**ace**	**Shannon H'**	**Simpson**
Day 0	610	125	303.14	575.37	41.23	21.73	3.07	0.21
NH4+CH4	527	2	2	2	100	100	0.04	0.99
NH4	5772	19	30.25	31.25	62.81	60.80	0.50	0.85
NO3+CH4	5072	7	7.5	8.79	93.33	79.67	0.70	0.65
NO3	1296	8	8.33	11.89	96.00	67.30	0.55	0.77
Day 28	448	146	411	760.30	35.52	19.20	3.51	0.11

*The calculations are based on 454 pyro sequencing reads and the data was normalized to 448 reads per sample*.

* *Calculated on data normalized to 448 random reads per sample*.

Based on pyro sequencing data 12 bacterial genera were present at over 1% relative abundance in the original groundwater. The most abundant bacteria belonged to an unassigned group of *Parcubacteria* (Rinke et al., 2012) [formerly Candidate division (Cd) OD1], the β-proteobacterial genus *Gallionella* and the *Microgenomates* (Rinke et al., 2012; formerly Cd OP11, Figure 3, Table S1). Interestingly, in the 28d incubation controls the relative abundance of *Sulfuricurvum* increased from 0 to 41.5%, the *Parcubacteria* group from 16.9 to 27.5% and the Comamonadaceae genus *Malikia* from 0 to 5.6%. In the NH_4_ and NH_4_+CH_4_ incubations *Malikia* contributed with 48.7% and 41.6% of the sequence reads, respectively, being the most abundant bacterial group enriched in these microcosms. *Methylobacter* was the second most abundant bacterial genus contributing with 35.9 and 43.8% of the sequence reads, respectively. In addition, *Hydrogenophaga* had become enriched (11.2% of the sequence reads) in the NH_4_+CH_4_ incubations but was present at below 1% in the NH_4_ microcosms. The NO_3_- and NO_3_+CH_4_ amendments enriched the *Malikia* most and 86.5% and 69.2% of the sequence reads belonged to this genus, respectively. In addition, 5.0% and 20.5% of the sequence reads belonged to *Sulfuricurvum* and 4.4% and 2.6% belonged to *Sulfuricella*, respectively. *Methylobacter* contributed with 2.4% of the sequence reads in the NO_3_+CH_4_—amended microcosms, but were not found in the microcosms with only NO_3_. Instead, the NO_3_—amended microcosms contained *Acetobacterium* sequences, 2.8%, which were additionally only detected in the original groundwater samples.

**Figure 4:**
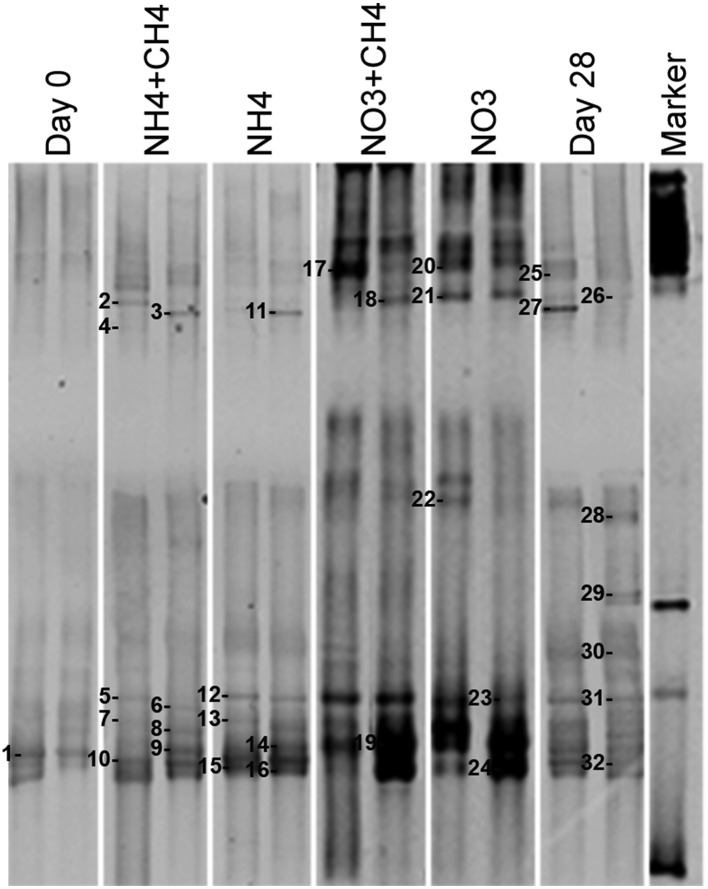
**Bacterial community profiles from PCR-DGGE of DNA extracted from original groundwater (Day 0) and supplemented vs. control microcosms (Day 28)**. The supplemented treatments are shown as described in Figure 2.

**Figure 5:**
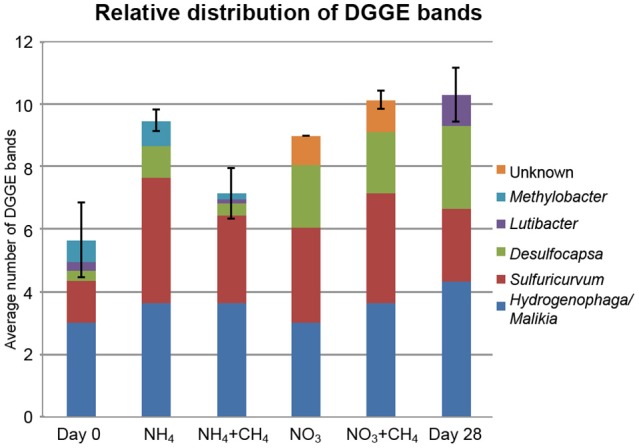
**Average number of DGGE bands per replicate treatment (X axis) and the distribution of bacterial taxa identified from the DGGE profiles of the different treatments (Y axis)**. The number of replicates was 3 (Day 0 and 28) or 6 per treatment (substrate supplemented microcosms). The error bars show standard error of mean (SEM). The supplemented treatments are shown as described in Figure 2.

**Figure 6:**
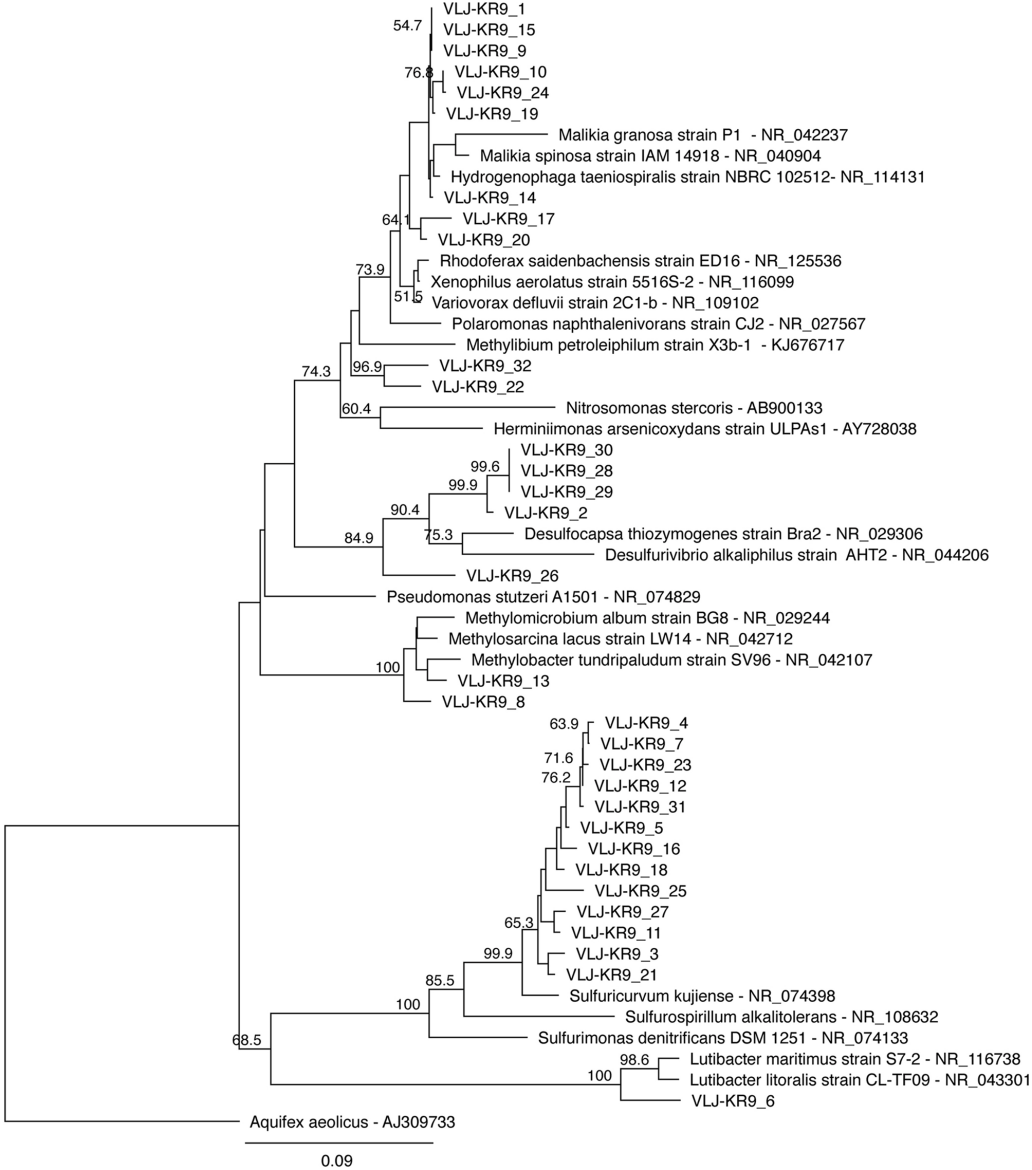
**A maximum likelihood tree displaying the sequences obtained from the 16S rRNA gene DGGE bands**. The sequences of this study are designated with the identifier VLJ-KR9 and the number at the end of the identifier describes the isolated DGGE band, corresponding to the bands in Figure 4. Bootstrap support for nodes was calculated with 1000 random repeats; nodes with more than 50% support are indicated.

The DGGE profiles showing the most abundant taxa in the bacterial communities were similar to the pyro sequencing profiles. The DGGE profiles of the bacterial taxa enriched during incubation were also similar between the different treatments, but specific phylotypes were more clearly detected in comparison to the ones seen in the original groundwater (Figure 4). The genera detected by DGGE affiliated with the β-proteobacterial *Hydrogenophaga/Malikia* cluster and ε-proteobacterial *Sulfuricurvum* (Figures 5, 6). The most common bacteria detected by DGGE in all substrate-amended microcosms belonged to ε-proteobacterial *Sulfuricurvum* and β-proteobacterial *Hydrogenophaga/Malikia* (Figures 5, 6), although the 454-pyrosequencing detected *Sulfuricurvum* enriched only in NO_3_+CH_4_—amended microcosms and in the 28d controls, and at low abundance in the NH_4_- and NO_3_-amended microcosms. DGGE bands belonging to γ-proteobacterial *Methylobacter* were found in the original groundwater and in the microcosms amended with NH_4_ and NH_4_+CH_4_ in the DGGE analysis (Figures 5, 6). In addition, δ-proteobacterial *Desulfocapsa* were detected by DGGE in all microcosms although they were not detected by 454 pyro sequencing.

#### Ammonia oxidizers

**Figure 7:**
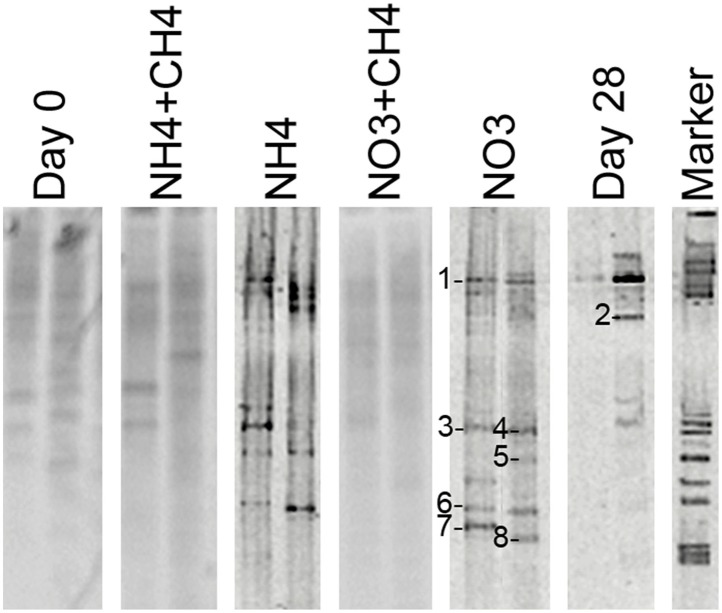
**Community profiles of PCR amplicons (***amo***A-targeted) from DNA of original groundwater (Day 0) and supplemented vs. control microcosms (Day 28)**. DGGE bands identified by sequencing are indicated by a number to the left of the band. The figure presents two replicate lanes out of 3 (Day 0 and Day 28) or 6 (NH_4_+CH_4_, NH_4_, NO_3_+CH_4_, NO_3_) replicate runs.

**Figure 8:**
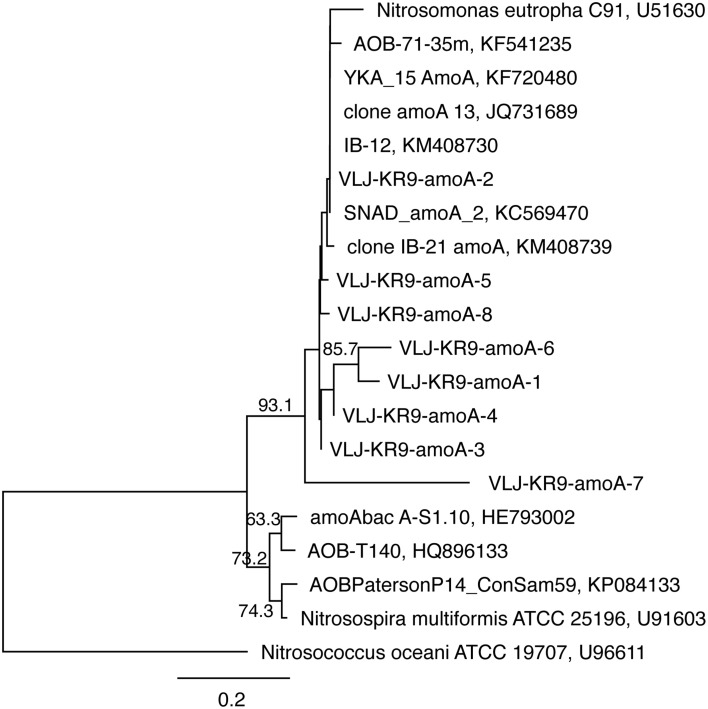
**The phylogenetic distribution of the ***amo***A fragments detected in this study presented as a maximum likelihood tree**. The gene sequences were translated into amino acid sequences prior to analysis. Bootstrap support for nodes was calculated with 1000 random repeats; nodes with more than 50% support are indicated. Sequences detected in this study are designated with the identifier VLJ-KR9-amoA; the number at the end of the identifier corresponds to the DGGE band indicated in Figure 7.

The *amo*A genes of ammonia oxidizing bacteria were detected by DGGE but of a total of 46 sequenced DGGE bands 8 bands provided a correct *amo*A sequence (Figure 7). Of these sequences, 7 were obtained from NO_3_-amended microcosms and one from unamended (control) microcosms. All detected *amo*A gene fragments were similar to β-proteobacterial *Nitrosomonas*-type *amo*A genes (Figure 8).

#### Nitrate reducers

**Figure 9:**
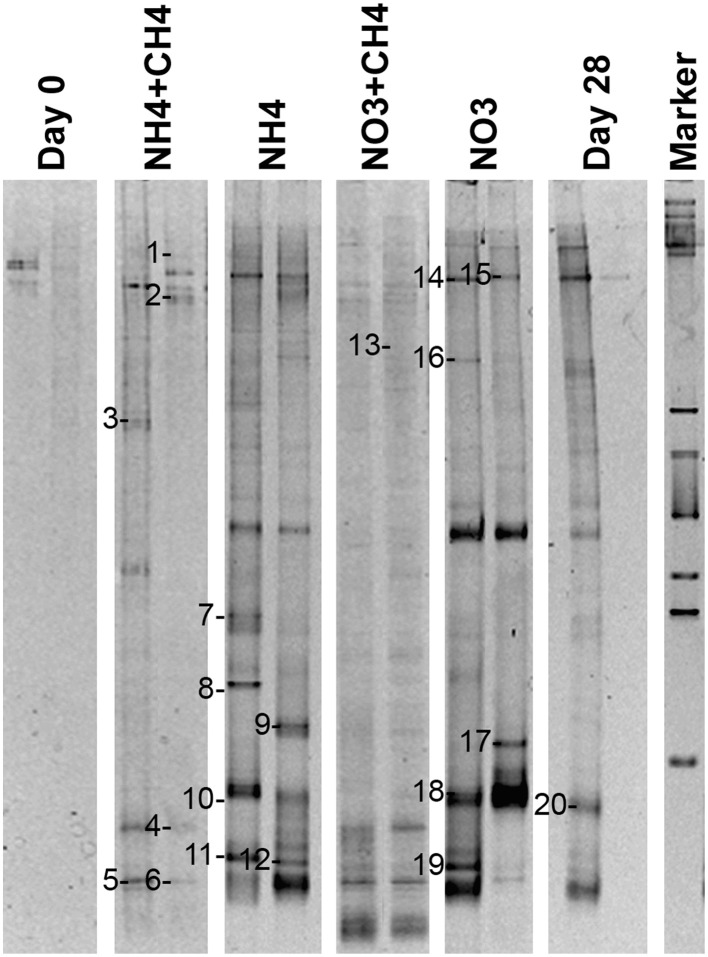
**Community profiles of PCR amplicons (***nar***G-targeted) from DNA of original groundwater (Day 0) and supplemented vs. control microcosms (Day 28)**. DGGE bands identified by sequencing are indicated by a number to the left of the band. The figure presents two replicate lanes out of 3 (Day 0 and Day 28) or 6 (NH_4_+CH_4_, NH_4_, NO_3_+CH_4_, NO_3_) replicate runs.

**Figure 10:**
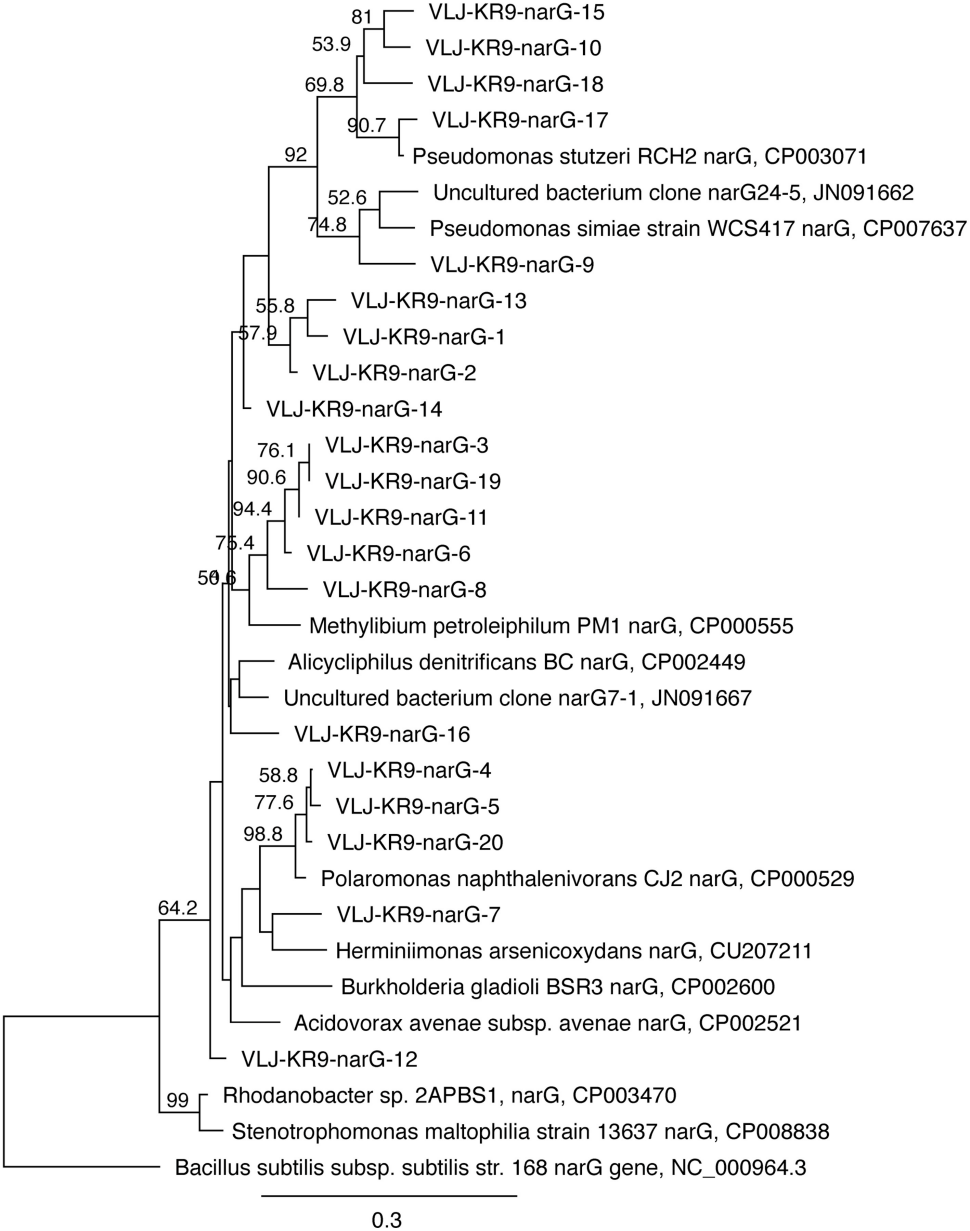
**The phylogenetic distribution of the ***nar***G fragments detected in this study presented as a maximum likelihood tree**. The gene sequences were translated into amino acid sequences prior to analysis. Bootstrap support for nodes was calculated with 1000 random repeats; nodes with more than 50% support are indicated. Sequences detected in this study are designated with the identifier VLJ-KR9-narG; the number at the end of the identifier corresponds to the DGGE band indicated in Figure 9.

*nar*G genes of NRB were detected by DGGE (Figure 9). Altogether 20 bands were successfully sequenced and identified to be *nar*G gene fragments. *nar*G gene sequences belonging to four different taxonomical groups were found (Figure 10). These sequences were similar to *narG* genes of *Pseudomonas* (bands 1, 2, 9, 10, 13, 14, 15, 17, 18), *Methylibium* (bands 3, 6, 8, 11, 19), *Alicycliphilus* (band 16), *Polaromonas* (bands 4, 5, 20), *Herminiimonas* (band 7), and to an uncultured group of bacteria (band 12). *Pseudomonas* and *Methylibium nar*G genes were frequently detected in all substrate-amended microcosms, while *Polaromonas nar*G was found in microcosms amended with NH_4_+CH_4_ or NH_4_ and in the untreated controls. *Alicycliphilus nar*G was detected only in microcosms supplemented with NO_3_ and only in one band. *nar*G band 12 was obtained from NH_4_ supplemented microcosms only, but the position of the DGGE band was similar to many others in the other treatments, which were not successfully sequenced; this indicated that the particular *nar*G type was common in the microcosms.

#### Nitrogen assimilating bacteria

The bacterial communities corresponding to different treatments changed over time due to the different substrates added to the microcosms. After double isopycnic centrifugation was applied in order to separate first the heavy DNA fraction from the light DNA fraction and then the ^15^N-enriched heavy DNA from the GC-rich heavy DNA, the amount of DNA left was not adequate to obtain DGGE bands of detectable density. However, 454 amplicon sequencing provided a low number of sequence reads from the ^15^N-enriched DNA, after pooling the three replicate microcosms within each treatment. Several genera belonging to α-proteobacterial Rhizobiales (*Rhizobium, Mesorhizobium, Tardiphaga*) and β-proteobacterial Burkholderiales (*Delftia, Undibacterium, Curvibacter, Pelomonas*) were detected in the ^15^N-enriched heavy DNA fraction from all amended microcosms (Figure 3; Table S1). In addition, sparse Nitrospirales 16S rRNA gene reads were found in the ^15^NH_4_-amended samples, along actinobacterial *Propionibacterium* from the ^15^NH_4_+CH_4_-amended microcosms (Table S1). In the ^15^NO_3_-amended samples, a few reads resembling cyanobacterial sequences were detected, and in the ^15^NO_3_+CH_4_-amended microcosms, an unassigned group of Armatimonadetes constituted the single most abundant 16S rRNA gene type detected in this treatment (Figure 3; Table S1). The corresponding fractions of the ^14^N substrate-amended microcosms were also tested by PCR, but no PCR fragments were obtained.

## Discussion

Nitrogenous compounds, such as nitrate and ammonia may have great effect on the microbial activities in the deep geological repository for low and intermediate level radioactive waste that has been constructed in to the crystalline bedrock of Olkiluoto, Finland. Enhanced microbial activity may cause microbially induced corrosion of the metallic waste, as shown by Rajala et al. (2015b) and affect the long-term safety of the repository. In oligotrophic crystalline bedrock environments methane may be an important component of gases dissolved in the groundwater and may serve as carbon source for the microbial communities and thus increase microbial activity. Nitrate-reducing microbial communities have been found in Olkiluoto deep groundwater by Most Probable Number analysis to depths greater than 300 m (Pedersen, 2008). Olkiluoto groundwater has in general large amounts of dissolved N_2_ and the amount of CH_4_ increases with depth from 1 to 1000 μL L^−1^ groundwater to a depth of 300 m, but increases 100-fold after this depth from where it may diffuse in to more shallow groundwater (e.g., Pedersen, 2008). Different N compounds may be introduced to the repository environment from explosives that have been incompletely detonated during construction of the repository.

In this study, we showed that added nitrate and ammonium had a great effect on the bacterial community in brackish groundwater in Olkiluoto at the depth of ca. 100 m. The bacterial community increased during the 28d incubation as a result amendment with N substrates and methane with the highest increase in the NH_4_+CH_4_ and NO_3_ treatments. The microbial community consumed NH_4_ during the first 2 weeks of incubation, both with and without CH_4_, but the consumption decreased toward the end of the incubation period. NO_3_ consumption was detected at the end of the incubation period, but not together with CH_4_. This indicates that the NH_4_- and NO_3_-consuming populations were not in general affected by CH_4_. However, the CH_4_-consuming community despite their intrinsic differences may affect the N-cycling community. The N substrates also decreased the number of detected OTUs, the estimated OTU richness and diversity indices of the microbial communities in the microcosms, while in the unamended 28d control microcosms these factors increased (Table 2). It is thus possible that introduction of N substrates in the repository area may have significant effect on the corrosion rate of the metallic radioactive waste by explicitly increasing specific types of microorganisms.

ε-proteobacteria belonging to the *Sulfuricurvum* sp. were found to be a main constituent of the bacterial communities in all microcosms according to the DGGE (Figure 5) and the pyro sequencing assay showed that their relative abundance clearly increased in the NO_3_- and NO_3_+CH_4_-amended microcosms and in the 28d controls (Figure 3; Table S1). These bacteria are common inhabitants of groundwater environments (Kodama and Watanabe, 2004; Bomberg et al., 2015) and it has recently been shown that *Sulfuricurvum denitrificans* use nitrate as electron acceptor. However, some species lack the common cytoplasmic membrane bound *Nar* nitrate reductase (Handley et al., 2014). In addition, Sievert et al. (2008) speculated that due to the lack of genes for nitrite ammonification (DNRA), *S. denitrificans* would have to incorporate ammonia from the environment. However, our results did not show any ε-proteobacterial enrichment in the ^15^N DNA fraction in any of the treatments, indicating that they did not assimilate N from any N-substrates used here. Nevertheless, it should be noted that we obtained very few sequences (24–180 reads per sample) from the ^15^N-DNA fraction over all, and if ε-proteobacteria incorporated only low amounts of labeled N they may well have gone undetected in this assay. It is also possible that *Sulfuricurvum* sp. does not use nitrate of ammonium as N source, but only as energy source.

Bacterial taxa affiliating with the *Hydrogenophaga*/*Malikia* cluster (Figure 6) was another group that appeared to benefit from N-substrate supplementations, according to the DGGE analyses. In the 454 pyro sequencing *Hydrogenophaga*/*Malikia* bacteria were not detected from the original groundwater, but were the dominating bacteria found in all N-substrate amended microcosms (49.6–86.5% of the sequence reads) and their relative abundance in the 28d microcosms also increased (Table S1). *Hydrogenophaga* and *Malikia* species reduce nitrate to nitrite (Spring et al., 2005; Yoon et al., 2008). In addition, *Hydrogenophaga* are able to perform the DNRA process thus producing ammonia from nitrate (Yoon et al., 2008). However, as with *Sulfuricurvum* sp., *Hydrogenophaga/Malikia* sequences were undetectable in the ^15^N-DNA fraction and may thus use other N sources that nitrate or ammonia.

*Methylobacter* sp. were found in all microcosms except the NO_3_—amended ones according to the pyro sequencing profiles and in all microcosms that received NH_4_+CH_4_, NH_4_, and NO_3_+CH_4_, i.e., in response to NH_4_ and CH_4_ according to DGGE (Table S1, Figure 5). Their relative abundance in the NH_4_+CH_4_– and NH_4_–amended microcosms was 43.8 and 35.9% (Figure 3), respectively, and below 2.4% in the other treatments. In general, ammonia has been considered to inhibit methanotrophs. However, methanotrophic bacteria may oxidize ammonium to hydroxylamine using methane monooxygenase (Hanson and Hanson, 1996; Campbell et al., 2011) and in accordance with our results, Zhang et al. (2014) showed that the abundance of *Methylobacter* increased in landfill cover soil as a result of increased NH_4_-N fertilization. Interestingly, Nyerges and Stein (2012) showed that ammonium had a distinct competitive effect on the oxidation of methane by methanotrophs.

The abundance of NRB increased in the microcosms during the incubations (Figure 2B). However, the ratio of *nar*G gene to bacterial 16S rRNA gene concentration was generally low. In addition to the membrane bound NAR enzyme many bacteria have a cytoplasmic NAP enzyme. Genes for this enzyme were not targeted in this study, i.e., we could detect only a part of the nitrate-reducing community. The detected *nar*G genes of NRB belonged to γ-proteobacterial *Pseudomonas* and β-proteobacterial *Methylibium, Alicycliphilus, Polaromonas, Herminiimonas*, and uncultured β-proteobacteria. *Methylibium* 16S rRNA gene sequences were not found in any treatment with either of the community profiling methods, although other Burkholderiales clades were detected. In contrast, *Pseudomonas* 16S rRNA genes or those of closely related groups were not found at all although *Pseudomonas* has been shown to be a major bacterial group at greater depth in Olkiluoto (Bomberg et al., 2015). *Polaromonas nar*G genes were detected in the NH_4_+CH_4_—amended and 28d control microcosms and similar DGGE bands were also faintly seen in most other samples. This is in accordance with the 454 pyro sequencing, which detected *Polaromonas* 16S rRNA gene sequences from all samples with the exception of the NH_4_+CH_4_—amended microcosms. It is possible that the *Polaromonas* escaped detection because the *Hydrogenophaga/Malikia* sequences were so dominating in this sample and the overall number of sequences obtained from this sample was low. *Herminiimonas na*rG were found only in the NH_4_—amended microcosms, but their 16S rRNA gene sequences were detected in the NO_3_+CH_4_—amended microcosms. This is not surprising, because even in the sample where *Herminiimonas* was detected they were present at only 0.04% relative abundance (Table S1) indicating that they belong to the minority groups at this depth in Olkiluoto. The only N assimilating bacterial group for which a functional gene was also detected was the *Alicycliphilus*. *Alicycliphilus nar*G was detected in the NO_3_—amended microcosms, but *Alicycliphilus* 16S rRNA genes were detected in the NH_4_+CH_4_^15^N SIP fraction. It is possible that this group was present in the other treatments as well, but failed detection due to the overall low number of sequence reads obtained from the ^15^N-DNA fractions.

The *nar*G gene specific primers we used here appear to have higher affinity to β- and γ-proteobacterial *nar*G genes than to those of the α- and ε-proteobacteria, which may lead to the α- and ε-proteobacteria being under-represented in this study (Nicolaisen and Ramsing, 2002). In addition, different primers were used for the *nar*G qPCR and *nar*G DGGE analyses, which may affect the detection efficiency to different *nar*G genes. In the *nar*G-qPCR assay the size of the produced amplicon was optimized for qPCR and was only 90 bp long. In order to increase sequence length a primer pair producing a 309 bp long amplicon was chosen for the *nar*G DGGE. However, the 90 bp long amplicon produced in the qPCR with primers *nar*G-1960m2f/*nar*G-2050m2F (López-Gutiérrez et al., 2004) have been shown to detect similar *nar*G sequences in Fennoscandian bedrock groundwater (Purkamo et al., 2015; Rajala et al., 2015a) as those detected with primers *nar*G-2179F/*nar*G2488R (Pastorelli et al., 2013) in this study.

*amo*A gene sequences were only obtained from the microcosms supplemented with NO_3_ and from the unamended 28d controls and the rest of the sequences were unspecific. All *amo*A genes were similar to those of *Nitrosomonas* bacteria. Interestingly, *Nitrosomonas* 16S rRNA genes were not detected by DGGE or 454 pyro sequencing in any of the samples. However, other closely related Gallionellales groups were detected in all other treatments except the NH_4_+CH_4_—amended microcosms (Table S1), and the *amo*A genes between these groups may be similar to that of the *Nitrosomonas*. The number of AOB in deep crystalline bedrock groundwater overall is expected to be low, which was shown previously by Purkamo et al. (2015). The fact that *amo*A genes were only successfully detected in the NO_3_-amended and 28d control microcosms is curious. It is, however, possible that the DGGE assay for *amo*A genes was not suitable for our sample matrix. We experienced some issues with the specificity of the primers used and in addition the DGGE bands obtained may have consisted of a mixture of amplicons. In the microcosms from which *amo*A gene fragments were successfully identified the number of interfering amplicons was probably lower and thus allowing us to detect clean sequences of the gene. Nevertheless, the AOB detected in this study in the NO_3_-amended and 28d control microcosms may have benefited from the slowly released lower concentration ammonia in the NO_3_—amended microcosms originating from the conversion of nitrate to ammonia through the DNRA process. In addition, it may also be possible that instead of typical ammonia oxidizers methanotrophic bacteria able to oxidize ammonium using their methane monooxygenase (Hanson and Hanson, 1996; Campbell et al., 2011) increased, and this group would not be detected by the *amo*A-gene targeted assays.

In the N-assimilating community, bacteria belonging to Rhizobiales genera were the most abundantly detected and were found in the ^15^N DNA fraction in all of the treatments (Figure 3, Table S1). Rhizobiales represented 72 and 61% of the N-assimilating bacterial community in the in the NH_4_- and NO_3_-amended microcosms. In the microcosms that received CH_4_ in addition to the N-substrates, their relative abundance in the N-assimilating community was lower, 4 and 35% in the NH_4_+CH_4_and NO_3_+CH_4_ treatments, respectively. In addition to Rhizobiales types, Burkholderiales were detected in the ^15^N DNA fraction of all treatments, but was especially abundant in the NH_4_+CH_4_ incubations. Interestingly, in the original groundwater approximately 8% of the 16S rRNA gene sequence reads obtained by 454 sequencing belonged to Rhizobiales, while by the end of the incubation period Rhizobiales-related sequence reads decreased to <0.5%.

The pyro sequencing and DGGE assays used in this study generally produced similar profiles of the dominating bacterial taxa in the sample although the pyro sequencing provided much more detail as well as abundance information. While DGGE is a visual method for fast detection of microbial community profiles and is excellent for comparison of microbial community development between a low number of samples, it has some restrictions. It could be concluded from e.g., the present work and from other groundwater studies (e.g., Bomberg et al., 2014) that the method generally only presents the main groups of the community. In addition, many microbial groups dominating in a specific environment have been shown to go undetected by the DGGE method due to their incompatibility with the method of detection, low amounts of target template or high level of intra-species microdiversity, which makes optimization of the DGGE conditions very demanding (e.g., Costa et al., 2006; Alonso-Sáez et al., 2007; Sánchez et al., 2009). This was the case also here, where the DGGE failed to detect e.g., the *Parcubacteria* present at high relative abundance in both the original groundwater and the 28d incubation control (Table S1). However, the pyro sequencing approach failed to detect *Sulfuricurvum* in the original groundwater and the NH_4_+CH_4_-supplemented microcosms, although they were found in all samples according to the DGGE analysis.

The primers chosen for a study may also greatly affect the results. In this study we showed that microbial profile results depend on the detection method and primers used for characterizing microbial communities. For example the primers U968fGC/U1401r (Nübel et al., 1996) used for DGGE detected *Desulfocapsa* in the 28d incubation controls, but the primers 8F (Edwards et al., 1998) and P2 (Muyzer et al., 1993) used for 454-pyrosequencing did not.

## Conclusions

The bacterial community of groundwater from 100 m depth in the VLJ-cave situated in the crystalline bedrock of the Fennoscandian Shield was affected by ammonia and nitrate supplementation. The bacterial population increased when N compounds were available, enriching specific bacterial groups. Known NRB belonging to ε-proteobacterial *Sulfuricurvum* and β-proteobacterial *Hydrogenophaga/Malikia* clades were greatly enriched when groundwater microcosms were supplemented with ammonium or nitrate with or without methane. *Methylobacter* was enriched in microcosms that had received ammonium with and without the addition of methane, indicating that the *Methylobacter* may also oxidize ammonia. In addition, it was demonstrated that bacterial clades belonging to Rhizobiales and Burkholderiales from nitrogen-poor deep crystalline bedrock groundwater were able to assimilate N from added nitrate and ammonium substrates. Here, the study of the N cycle in crystalline bedrock groundwater was restricted to specific enzymes of specific bacterial groups. In future investigations the N cycling genes of different taxonomic groups as well as different enzymes, such as the soluble nitrate reductase NAP, should be included. It would also be of value to investigate the N_2_ fixation and anaerobic ammonia oxidation (ANAMMOX) and archaeal ammonia oxidation in order to obtain a complete picture of the N cycle in deep crystalline bedrock environments. Nevertheless, we show here that despite the low abundance of N cycling bacteria in the pristine groundwater of Olkiluoto, N assimilating, ammonia oxidizing and nitrate reducing bacteria increase when the concentration of ammonia and nitrate increase, which may influence the long-term safety of the underground repository for low and intermediate level radioactive waste.

## Author contributions

HK, PR, LC, and MB conceived, designed and performed the experiments, analyzed the data and wrote the paper.

## Funding

The work was performed on funding provided by the Finnish Research Program on Nuclear Waste Management 2011–2014 (project MICCU), and the Academy of Finland (project 261220).

### Conflict of interest statement

The authors declare that the research was conducted in the absence of any commercial or financial relationships that could be construed as a potential conflict of interest.
